# SIRT1 performs a balancing act on the tight-rope toward longevity

**DOI:** 10.18632/aging.100076

**Published:** 2009-07-30

**Authors:** Aparna Purushotham, Thaddeus T. Schug, Xiaoling Li

**Affiliations:** Laboratory of Signal Transduction, National Institute of Environmental Health Sciences, National Institutes of Health, Research Triangle Park, NC 27709, USA; ^1^ These authors contributed equally to this work

**Keywords:** SIRT1, PPARα, hepatic fatty acid oxidation, PGC-1α, gluconeogenesis

## Abstract

Our recent study defined a new role for SIRT1 as a regulator of hepatic lipid metabolism. In the liver a major target of this sirtuin is the PPARα/PGC-1α signaling axis. Ablation of SIRT1 in the liver results in disrupted fatty acid oxidation, increased cellular stress, and elevations in proinflammatory cytokines. However, contrary to previous studies, we observed no changes in glucose production in the absence of SIRT1, despite impaired PGC-1α signaling. These findings point toward the involvement of other players in SIRT1-regulated hepatic metabolism. Here we discuss our findings, and comment on some of the controversy surrounding this protein in the current literature.

The food we eat has long been linked to the rate we age. Selective pressures in times of food abundance and scarcity have influenced our very genetic makeup, instilling in our genome genes believed to control the delicate balance between metabolism and aging. However, this balance has been disrupted in western societies with developments in agriculture and technologies that have promoted the intake of high-calorie diets and sedentary lifestyles. We are witnessing an alarming increase in the rate of metabolic syndrome, which consists of a collection of abnormalities including obesity, type 2 diabetes, dyslipidemia, fatty liver, and a pro-inflammatory and prothrombotic state [1,2] Currently, one in four adults in the United States suffers from metabolic syndrome and worldwide estimates are over 2.1 billion [3,4]. Ultimately, this epidemic threatens human life-span projections and puts great pressure on our already overburdened health care system.

The sirtuin family of proteins appears to be at the crossroads between nutritional status and longevity. Sirtuins are highly conserved NAD^+^-dependent protein deacetylases and/or ADP ribosyltransferases that target histones, transcription factors, and co-regulators to adapt gene expression in response to the cellular energy state [5]. Many members of this family, including the founder Sir2, have been shown to impact aging in species ranging from yeast to fly and it is believed these protective actions result from the beneficial regulation of stress management, and energy homeostasis. SIRT1, the mammalian ortholog of Sir2, plays a role in numerous physiological processes including fat metabolism, glucose homeostasis and immune response. Because SIRT1 activity is dependent on the energy status of the cell, it provides a direct link between metabolism, chromosome structure, and metabolic gene regulation [6].

The liver is a central metabolic organ in charge of regulating nutrient homeostasis in fed and fasting conditions. It controls key aspects of lipid and glucose metabolism in response to nutritional and hormonal signals [7]. Tight regulation of glucose by the liver is essential to ensuring that glucose-dependent tissues such as brain and red blood cells have ample energy supply during periods of nutrient deprivation. Recent reports have shown that SIRT1 protein levels and enzymatic activity are induced in the fasted liver [8,9]. SIRT1 regulates genes involved in gluconeogenesis through deacetylation of several key transcription factors and coactivators [8,9,10]. The liver also plays an important role in maintaining lipid homeostasis. In line with its role as a metabolic mediator, SIRT1 is known to regulate genes involved in fatty acid oxidation and lipolysis [11]. Interestingly, the SIRT1 activator resveratrol has shown promise as a therapeutic agent for the treatment of metabolic diseases [12,13]. Mice fed a high-fat diet along with resveratrol remained lean and healthy compared to over-weight control animals [13]. Additionally, resveratrol significantly increased aerobic capacity, as evidenced by increased running time and elevated oxygen consumption in muscle fibers. Resveratrol treatment also protected mice against diet-induced-obesity and insulin resistance [12]. Groups are now focusing on the development of high affinity small molecule activators of SIRT1 as a therapeutic approach for treating diseases of aging such as type-2 diabetes [14].

Although SIRT1 is an important regulator of metabolism, the tissue-specific and systemic roles of SIRT1 are difficult to dissect *in vivo*, primarily due to the complicated developmental defects in the SIRT1 whole-body knockout mouse [15,16]. In search of further evidence to identify a tissue-specific role of SIRT1 in the regulation of energy homeostasis, we developed a knockout mouse model containing hepatic deletion of SIRT1 (LKO) [17]. Microarray analysis of liver from LKO mice revealed a striking reduction in expression of genes regulated by the peroxisome proliferators-activated receptors α (PPARα). This lipid sensing nuclear receptor is an important mediator of the adaptive response to fasting and starvation.  Deletion of SIRT1 in the liver impairs PPARα signaling and decreases fatty acid β-oxidation, whereas over-expression of SIRT1 induces expression of PPARα target genes. Furthermore, we found that SIRT1 regulates PPARα signaling by directly interacting with the PPARα nuclear receptor. This interaction appears to be ligand dependent, as SIRT1 is recruited to response elements on promoters of PPARα target genes by agonists as well as by changes of nutritional status. One mechanism by which SIRT1 regulates PPARα signaling in the liver appears to be through the hands of PGC-1α, a key coactivator for PPARα signaling and a direct target of SIRT1 [9,18]. It has been shown that SIRT1 activates PGC-1α primarily by its deacetylation [9] (Figure 2). In keeping with these findings, we observed that although PGC-1α message levels are lower in SIRT1  LKO livers, PGC-1α protein accumulates on promoter regions of PPARα target genes but in a less active hyperacetylated form. These findings suggest that activated PGC-1α is required for promoting transcription of PPARα targets and that SIRT1 may be involved in monitoring the recruitment/dissociation cycle of PGC-1α. Additionally, GST-pull down mapping data showed that the core domain of SIRT1 directly interacts with PPARα. Therefore, another plausible mechanism underlying our observations is that PPARα may be a *bona fide* SIRT1 substrate. Further studies are necessary to elucidate weather SIRT1 indeed deacetylates PPARα, thereby affecting its activity.

**Figure 1.**
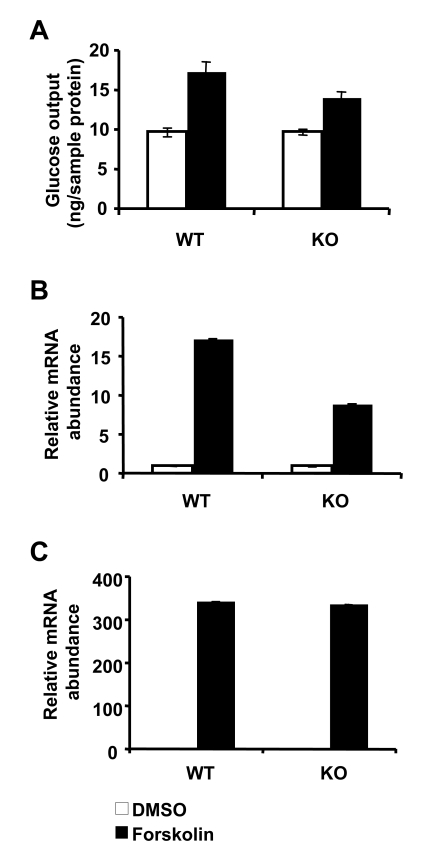
Loss of SIRT1 has minimal impact on gluconeogenesis in primary hepatocytes. (**A**) Glucose output from primary hepatocytes isolated from control and SIRT1 LKO mice. Cells were treated with DMSO (white bars) or 10 μM forskolin (black bars) and incubated for 6 h in glucose free DMEM supplemented with 20 mM sodium lactate and 2 mM sodium pyruvate. Glucose output was measured in culture medium using a glucose oxidase kit (Sigma). Data represent mean + SD.  (**B-C**) SIRT1 deficiency in primary hepatocytes reduces the induction of PGC-1α (**B**) but not PEPCK (**C**) message in response to 10μM forskolin treatment. mRNA from primary hepatocytes treated with DMSO (white bars) or forskolin (black bars) were analyzed using qPCR. Data represent mean + SD.

A major focus of our study was to characterize how disruptions in PPARα signaling affect the physiology of SIRT1 LKO mice [17]. When challenged with a high-fat diet, LKO mice displayed increased hepatic steatosis and hallmarks of endoplasmic reticulum stress and inflammatory responses. Interestingly, in a trend very similar to those reported in the PPARα knockout mouse, LKO mice displayed elevated levels of proinflammatory cytokines. These observations indicate that SIRT1 LKO mice are prone to development of hepatic inflammation, which has been implicated in the progression of insulin resistance [9,20]. These findings provide evidence that solidify SIRT1's role as a key regulator of metabolic homeostasis and complement previous animal studies using pharmacological tools [14] or modest SIRT1 overexpression mouse models [21,22].

Several of the metabolic abnormalities we observed in the SIRT1 LKO mice [17], however, are in direct contrast to those recently reported by Chen et al. [23].

**Figure 2.**
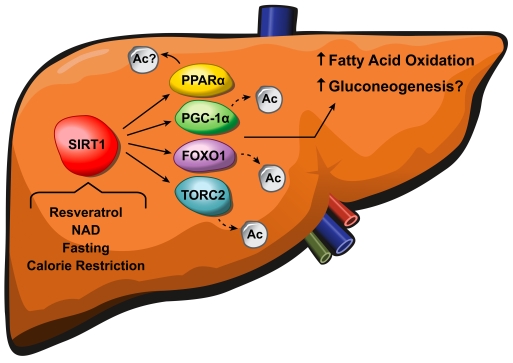
SIRT1 regulates fatty acid oxidation and gluconeogenesis in the liver. Resveratrol, NAD^+^, fasting and calorie restriction activate SIRT1, causing deacetylation of PGC-1α, FOXO1, and TORC2 which in turn leads to increased fatty acid oxidation and gluconeogenesis. The exact mechanism underlying how SIRT1 activates PPARα and the precise role of PGC-1α in the SIRT1-mediated glucose homeostasis remain to be clarified.

Using a similar hepatic-specific knockout mouse model, Chen et al. observed a reduction in weight gain and liver fat accumulation in LKO mice when fed a western-style diet. Additionally, their mice were protected from the physiological impacts of a western diet with lower blood glucose and insulin levels. Similar to our study, their group observed minor physiological differences in LKO mice fed a chow diet. In wake of these findings, Chen et al. proposed that SIRT1 activity in the liver is directly proportional to calorie intake, and that excess calories and/or SIRT1 activators may result in elevated synthesis of fat and cholesterol. One possible factor contributing to the discrepancy between our observations and those of Chen et al. may be the difference in age of animals at which the feeding was initiated and data were collected. In our study, mice were six-week old when high-fat diet feeding was initiated, whereas four-month old mice were utilized in the study carried out by Chen et al. The varied responses of SIRT1 LKO mice to a western-style diet at different ages raises the possibility that hepatic SIRT1 may selectively regulate alternative metabolic pathways at multiple stages of development. An inducible SIRT1 knockout model will be helpful to dissect age-dependent effects of SIRT1.  Moreover, since the liver is such a dynamic metabolic organ, small variations in dietetic components and genetic backgrounds may also contribute to the inconsistency between these two studies.

Another surprising phenotype observed in the SIRT1 LKO mice is their normal gluconeogenesis in response to a 16-h fasting [17]. The inducible coactivator PGC-1α is an important component of a number of transcriptional complexes that regulate glucose and lipid metabolism. Hepatic knockdown of SIRT1 significantly abrogates the fasting induction of gluconeogenic genes by regulating the acetylation status of PGC1α [11]. However, we observed no changes in fasting glucose levels in the absence of hepatic SIRT1 despite impaired PGC-1α signaling.  Liver specific SIRT1 knockout mice had slightly higher, although not statistically significant, fasting glucose levels compared to littermate controls upon high-fat feeding. Expression levels of the two rate-limiting enzymes in the gluconeogenic pathway, PEPCK and G-6Pase, were also unchanged in the absence of hepatic SIRT1. Consistent with these observations, forskolin, an intracellular cAMP stimulator, promoted gluconeogenesis independently of SIRT1 levels in primary hepatocytes (Figure 1A). Additionally, although the forskolin-mediated induction of PGC1α expression was decreased in these cells (Figure 1B), the overall message levels of PEPCK remained similar between control and LKO hepatocytes (Figure 1C). Gluconeogenesis is regulated by a complex interplay between transcription factor and hormonal and coregulator signaling. While PGC-1α is known to control hepatic glucose production, other factors such as FOXO1 and TORC2 are reported to promote gluconeo-genesis [24]. Interestingly, SIRT1 has been shown to deacetylate and repress both FOXO1 [25] and TORC2 [24]. Therefore, a likely explanation for our findings is that while PGC-1α activity is lower in SIRT1 KO livers, compensatory effects of FOXO1 and TORC2 balance the reduction in PGC-1α signaling (Figure 2). Another possible explanation for the contradiction in these studies may lie in differences in cell types and method of SIRT1 deletion/knockdown used in the animal studies. It is important to note that the hepatic-specific albumin-Cre driven SIRT1 knockout mouse utilized in our study is a permanent knockout model. Phenotypes observed in these mice may reflect systemic and local compensatory effects in wake of hepatic deletion of SIRT1. Studies done by Rodger et al. [11] employed transient knockdown methods using adenovirus-mediated shRNA which seem to provoke more acute responses to loss of hepatic SIRT1.

In conclusion, while our study defines a new role for SIRT1 as a key regulator of hepatic lipid metabolism, it also adds fuel to the fire of controversy surrounding this protein as a central player in mammalian energy homeostasis. It appears that in the liver, a major target of this sirtuin is the PPARα/PGC-1α signaling axis. Ablation of SIRT1 in the liver creates disruptions in fatty acid oxidation, increased cellular stress, and elevations in proinflammatory cytokines. What remains to be determined is the precise role SIRT1 plays in regulating gluconeogenesis and cholesterol metabolism in the liver and how this, in turn, affects systemic metabolism. Our findings and others suggest that activation of SIRT1 may provide a therapeutic strategy for treatment of metabolic syndrome.
